# *catena*-Poly[[[(2-pyridone-κ*O*)silver(I)]-μ-2-pyridone-κ^2^ *O*:*O*] hexa­fluorido­phosphate]

**DOI:** 10.1107/S1600536810035348

**Published:** 2010-09-04

**Authors:** Hadi D. Arman, Tyler Miller, Edward R. T. Tiekink

**Affiliations:** a Department of Chemistry, The University of Texas at San Antonio, One UTSA Circle, San Antonio, Texas 78249-0698, USA; b Department of Chemistry, University of Malaya, 50603 Kuala Lumpur, Malaysia

## Abstract

The asymmetric unit of the polymeric title salt, {[Ag(C_5_H_5_NO)_2_]PF_6_}_*n*_, comprises an Ag^I^ cation (located on a twofold axis), two 2-pyridone ligands (with distinct coordination modes), and half a PF_6_^−^ anion (situated on a centre of inversion). The Ag^I^ atom is in an approximately octa­hedral AgO_6_ coordination geometry, which is stabilized by intra­molecular N—H⋯O hydrogen bonds. The result of the bridging mode of the 2-pyridone ligand is the formation of a supra­molecular chain along the *c* axis; these are consolidated in the crystal by C—H⋯F inter­actions.

## Related literature

For structural diversity in the supra­molecular structures of silver salts, see: Kundu *et al.* (2010 ▶). For a related Ag structure, see: Arman *et al.* (2010 ▶).
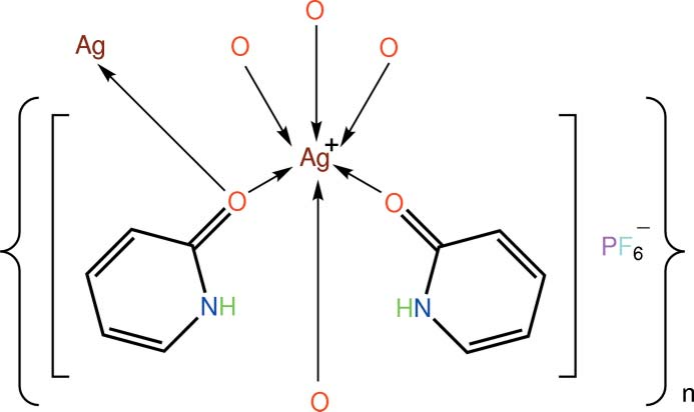


## Experimental

### 

#### Crystal data

[Ag(C_5_H_5_NO)_2_]PF_6_
*M* *_r_* = 633.24
Monoclinic, 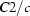

*a* = 13.519 (5) Å
*b* = 24.187 (9) Å
*c* = 7.301 (3) Å
β = 96.918 (5)°
*V* = 2369.9 (16) Å^3^
*Z* = 4
Mo *K*α radiation
μ = 1.00 mm^−1^
*T* = 293 K
0.48 × 0.40 × 0.14 mm

#### Data collection

Rigaku AFC12/SATURN724 diffractometer
Absorption correction: multi-scan (*ABSCOR*; Higashi, 1995 ▶) *T*_min_ = 0.535, *T*_max_ = 1.000
8382 measured reflections
2703 independent reflections
2573 reflections with *I* > 2σ(*I*)
*R*_int_ = 0.033

#### Refinement

*R*[*F* ^2^ > 2σ(*F* ^2^)] = 0.031
*wR*(*F* ^2^) = 0.080
*S* = 1.14
2703 reflections
165 parameters
H-atom parameters constrained
Δρ_max_ = 0.78 e Å^−3^
Δρ_min_ = −0.47 e Å^−3^

### 

Data collection: *CrystalClear* (Molecular Structure Corporation & Rigaku, 2005 ▶); cell refinement: *CrystalClear*; data reduction: *CrystalClear*; program(s) used to solve structure: *SHELXS97* (Sheldrick, 2008 ▶); program(s) used to refine structure: *SHELXL97* (Sheldrick, 2008 ▶); molecular graphics: *DIAMOND* (Brandenburg, 2006 ▶); software used to prepare material for publication: *publCIF* (Westrip, 2010 ▶).

## Supplementary Material

Crystal structure: contains datablocks global, I. DOI: 10.1107/S1600536810035348/hb5626sup1.cif

Structure factors: contains datablocks I. DOI: 10.1107/S1600536810035348/hb5626Isup2.hkl

Additional supplementary materials:  crystallographic information; 3D view; checkCIF report

## Figures and Tables

**Table 1:** Selected bond lengths (Å)

Ag—O1	2.3543 (19)
Ag—O2	2.5055 (18)
Ag—O2^i^	2.6278 (19)

Symmetry code: (i) 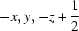.

**Table 2:** Hydrogen-bond geometry (Å, °)

*D*—H⋯*A*	*D*—H	H⋯*A*	*D*⋯*A*	*D*—H⋯*A*
N1—H1⋯O2	0.86	1.91	2.765 (2)	171
N2—H2⋯O1^ii^	0.86	1.90	2.754 (3)	174
C3—H3⋯F1^iii^	0.93	2.48	3.353 (3)	157
C5—H5⋯F3^iv^	0.93	2.51	3.398 (3)	159

Symmetry codes: (ii) 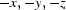; (iii) 
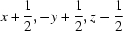
; (iv) 
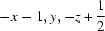
.

**Fig. 1.**
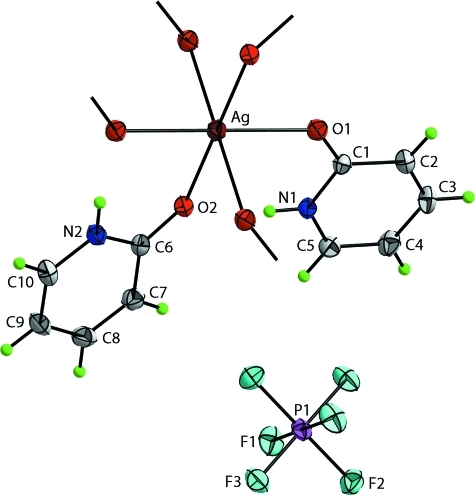
Asymmetric unit in the structure of (I) showing displacement ellipsoids at the 50% probability level. The Ag (lying on a 2-fold axis of symmetry) and P (lying on a centre of inversion) atom environments have been expanded to show the respective coordination geometries.

**Fig. 2.**
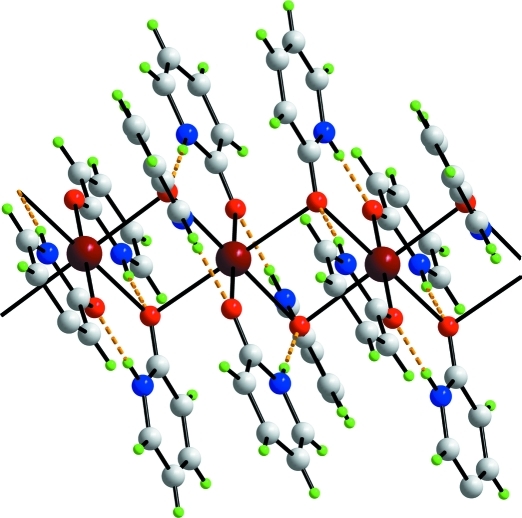
Portion of the supramolecular chain aligned along the c axis in (I).

**Fig. 3.**
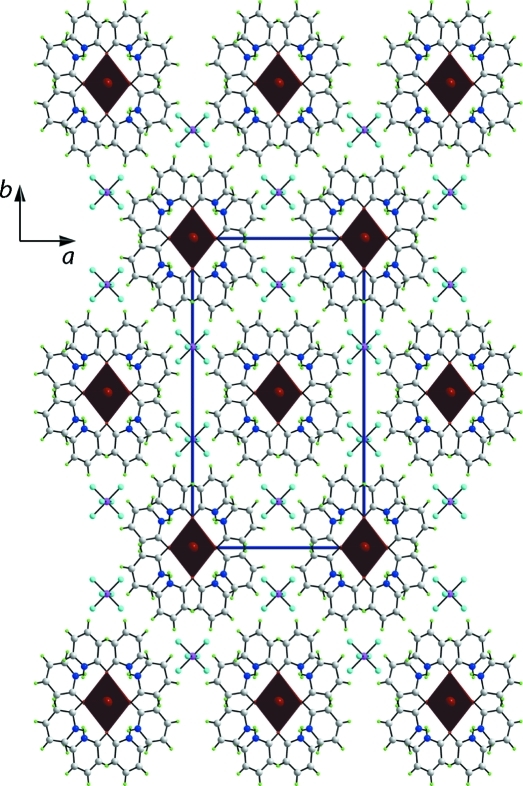
A view in projection down the c axis of the crystal packing in (I), emphasizing the Ag octahedra and interspersing of the PF6- anions.

## supplementary crystallographic information

### Comment

The structural diversity in the supramolecular structures of silver salts is well documented (Kundu *et al.*, 2010). The title compound, (I), was was isolated and characterized as a continuation of recent structural studies of such structures (Arman *et al.*, 2010).

The crystallographic asymmetric unit of (I) comprises half a Ag cation, situated on a crystallographic 2-fold axis, a monodentate 2-pyridone ligand, coordinating *via* the carbonyl-O atom, a bidentate 2-pyridone ligand, bridging two Ag cations *via* a carbonyl-O atom, and half a PF_6_^-^ anion, situated about a crystallographic centre of inversion, Fig. 1. The resulting Ag^I^ atom coordination geomerty is based on a distorted octahedron defined by an O_6_ donor set, with the Ag—O bond distances lying in the range 2.3543 (19) to 2.6278 (19) Å, Table 1. The coordination geometry is stabilized by intramolecular N—H···O hydrogen bonds, Table 2. As the carbonyl-O2 atom is bidentate bridging, a supramolecular chain along the *c* axis is generated, Fig. 2. The chains are consolidated in the 3-D structure by C—H···F interactions, Fig. 3.

### Experimental

The title salt, (I), was isolated as colourless blocks from the 1:2 reaction of silver hexafluorophosphate (Aldrich) and 2-hydroxypyridine (Aldrich) in methanol solution; m. pt 393–399 K.

### Refinement

The H-atoms were placed in calculated positions (N—H = 0.86 Å and C—H = 0.93 Å) and were included in the refinement in the riding model approximation with *U*_iso_(H) set to 1.2*U*_eq_(carrier atom).

### Crystal data

**Table:**

[Ag(C_5_H_5_NO)_2_]PF_6_	*F*(000) = 1264
*M**_r_* = 633.24	*D*_x_ = 1.775 Mg m^−^^3^
Monoclinic, *C*2/*c*	Mo *K*α radiation, λ = 0.71069 Å
Hall symbol: -C 2yc	Cell parameters from 4745 reflections
*a* = 13.519 (5) Å	θ = 3.1–40.6°
*b* = 24.187 (9) Å	µ = 1.00 mm^−^^1^
*c* = 7.301 (3) Å	*T* = 293 K
β = 96.918 (5)°	Block, colourless
*V* = 2369.9 (16) Å^3^	0.48 × 0.40 × 0.14 mm
*Z* = 4	

### Data collection

**Table:**

Rigaku AFC12K/SATURN724 diffractometer	2703 independent reflections
Radiation source: fine-focus sealed tube	2573 reflections with *I* > 2σ(*I*)
graphite	*R*_int_ = 0.033
ω scans	θ_max_ = 27.5°, θ_min_ = 3.1°
Absorption correction: multi-scan (*ABSCOR*; Higashi, 1995)	*h* = −14→17
*T*_min_ = 0.535, *T*_max_ = 1.000	*k* = −30→31
8382 measured reflections	*l* = −9→9

### Refinement

**Table:**

Refinement on *F*^2^	Primary atom site location: structure-invariant direct methods
Least-squares matrix: full	Secondary atom site location: difference Fourier map
*R*[*F*^2^ > 2σ(*F*^2^)] = 0.031	Hydrogen site location: inferred from neighbouring sites
*wR*(*F*^2^) = 0.080	H-atom parameters constrained
*S* = 1.14	*w* = 1/[σ^2^(*F*_o_^2^) + (0.036*P*)^2^ + 3.3853*P*] where *P* = (*F*_o_^2^ + 2*F*_c_^2^)/3
2703 reflections	(Δ/σ)_max_ = 0.001
165 parameters	Δρ_max_ = 0.78 e Å^−^^3^
0 restraints	Δρ_min_ = −0.47 e Å^−^^3^

### Special details

**Table:**

Geometry. All e.s.d.'s (except the e.s.d. in the dihedral angle between two l.s. planes) are estimated using the full covariance matrix. The cell e.s.d.'s are taken into account individually in the estimation of e.s.d.'s in distances, angles and torsion angles; correlations between e.s.d.'s in cell parameters are only used when they are defined by crystal symmetry. An approximate (isotropic) treatment of cell e.s.d.'s is used for estimating e.s.d.'s involving l.s. planes.
Refinement. Refinement of *F*^2^ against ALL reflections. The weighted *R*-factor *wR* and goodness of fit *S* are based on *F*^2^, conventional *R*-factors *R* are based on *F*, with *F* set to zero for negative *F*^2^. The threshold expression of *F*^2^ > σ(*F*^2^) is used only for calculating *R*-factors(gt) *etc*. and is not relevant to the choice of reflections for refinement. *R*-factors based on *F*^2^ are statistically about twice as large as those based on *F*, and *R*- factors based on ALL data will be even larger.

### Fractional atomic coordinates and isotropic or equivalent isotropic displacement parameters (Å^2^)

**Table:**

	*x*	*y*	*z*	*U*_iso_*/*U*_eq_	
Ag	0.0000	0.0000	0.0000	0.02327 (9)	
P1	−0.5000	0.14921 (3)	0.2500	0.02229 (17)	
O1	0.00398 (11)	0.09711 (7)	−0.0200 (2)	0.0243 (3)	
N1	−0.13089 (13)	0.11598 (7)	0.1275 (2)	0.0206 (3)	
H1	−0.1378	0.0811	0.1457	0.025*	
F1	−0.52808 (12)	0.14954 (6)	0.4566 (2)	0.0349 (3)	
O2	−0.13415 (13)	0.00438 (6)	0.2083 (2)	0.0239 (3)	
C6	−0.19943 (16)	−0.03274 (9)	0.2224 (3)	0.0214 (4)	
N2	−0.18133 (13)	−0.08533 (7)	0.1662 (2)	0.0223 (4)	
H2	−0.1265	−0.0916	0.1210	0.027*	
C1	−0.05410 (15)	0.13279 (8)	0.0342 (3)	0.0195 (4)	
F2	−0.58219 (11)	0.19640 (6)	0.1951 (2)	0.0340 (3)	
F3	−0.58231 (11)	0.10246 (6)	0.1955 (2)	0.0356 (3)	
C5	−0.19677 (17)	0.15108 (9)	0.1932 (3)	0.0240 (4)	
H5	−0.2478	0.1371	0.2544	0.029*	
C10	−0.24535 (18)	−0.12841 (9)	0.1781 (3)	0.0261 (4)	
H10	−0.2291	−0.1632	0.1369	0.031*	
C2	−0.04687 (17)	0.19098 (9)	0.0059 (3)	0.0237 (4)	
H2A	0.0028	0.2048	−0.0591	0.028*	
C3	−0.11206 (18)	0.22646 (9)	0.0732 (3)	0.0279 (5)	
H3	−0.1059	0.2643	0.0548	0.033*	
C7	−0.29123 (17)	−0.02432 (10)	0.2950 (3)	0.0263 (5)	
H7	−0.3080	0.0108	0.3333	0.032*	
C9	−0.33255 (17)	−0.12096 (10)	0.2493 (3)	0.0293 (5)	
H9	−0.3762	−0.1503	0.2585	0.035*	
C4	−0.18849 (18)	0.20657 (9)	0.1700 (3)	0.0283 (5)	
H4	−0.2324	0.2308	0.2171	0.034*	
C8	−0.35494 (17)	−0.06760 (11)	0.3087 (3)	0.0296 (5)	
H8	−0.4142	−0.0616	0.3584	0.036*	

### Atomic displacement parameters (Å^2^)

**Table:**

	*U*^11^	*U*^22^	*U*^33^	*U*^12^	*U*^13^	*U*^23^
Ag	0.02195 (14)	0.01698 (14)	0.03202 (15)	0.00192 (7)	0.00785 (10)	0.00250 (8)
P1	0.0220 (4)	0.0201 (4)	0.0267 (4)	0.000	0.0106 (3)	0.000
O1	0.0232 (8)	0.0203 (8)	0.0304 (8)	0.0016 (5)	0.0076 (6)	0.0000 (6)
N1	0.0225 (8)	0.0173 (8)	0.0225 (8)	0.0008 (6)	0.0045 (7)	0.0029 (7)
F1	0.0418 (8)	0.0363 (8)	0.0298 (7)	0.0041 (7)	0.0174 (6)	0.0042 (6)
O2	0.0225 (8)	0.0191 (8)	0.0308 (9)	−0.0005 (5)	0.0063 (7)	0.0036 (6)
C6	0.0217 (10)	0.0204 (10)	0.0220 (10)	0.0003 (8)	0.0023 (8)	0.0040 (8)
N2	0.0218 (8)	0.0215 (9)	0.0244 (9)	−0.0021 (7)	0.0062 (7)	0.0015 (7)
C1	0.0198 (9)	0.0201 (10)	0.0186 (9)	−0.0001 (7)	0.0019 (7)	0.0017 (7)
F2	0.0328 (8)	0.0300 (7)	0.0408 (8)	0.0092 (6)	0.0106 (6)	0.0067 (6)
F3	0.0307 (7)	0.0292 (7)	0.0480 (9)	−0.0084 (6)	0.0086 (6)	−0.0009 (6)
C5	0.0239 (10)	0.0258 (11)	0.0228 (10)	0.0027 (8)	0.0055 (8)	0.0020 (8)
C10	0.0327 (11)	0.0213 (10)	0.0245 (10)	−0.0065 (9)	0.0043 (9)	0.0013 (8)
C2	0.0277 (11)	0.0197 (10)	0.0239 (10)	−0.0026 (8)	0.0039 (8)	0.0021 (8)
C3	0.0364 (12)	0.0165 (10)	0.0311 (11)	0.0019 (9)	0.0056 (10)	0.0018 (8)
C7	0.0234 (10)	0.0283 (12)	0.0278 (11)	0.0032 (8)	0.0048 (9)	0.0021 (9)
C9	0.0272 (11)	0.0320 (12)	0.0286 (11)	−0.0115 (9)	0.0025 (9)	0.0052 (9)
C4	0.0316 (12)	0.0234 (11)	0.0312 (11)	0.0070 (9)	0.0092 (9)	−0.0008 (9)
C8	0.0208 (10)	0.0406 (14)	0.0278 (11)	−0.0022 (9)	0.0042 (9)	0.0049 (10)

### Geometric parameters (Å, °)

**Table:**

Ag—O1^i^	2.3543 (19)	C6—C7	1.422 (3)
Ag—O1	2.3543 (19)	N2—C10	1.364 (3)
Ag—O2^i^	2.5055 (18)	N2—H2	0.8600
Ag—O2	2.5055 (18)	C1—C2	1.427 (3)
Ag—O2^ii^	2.6278 (19)	C5—C4	1.359 (3)
Ag—O2^iii^	2.6278 (19)	C5—H5	0.9300
P1—F1	1.5993 (15)	C10—C9	1.356 (3)
P1—F1^iv^	1.5993 (15)	C10—H10	0.9300
P1—F3^iv^	1.6026 (15)	C2—C3	1.363 (3)
P1—F3	1.6026 (15)	C2—H2A	0.9300
P1—F2^iv^	1.6095 (15)	C3—C4	1.405 (3)
P1—F2	1.6095 (15)	C3—H3	0.9300
O1—C1	1.262 (3)	C7—C8	1.367 (3)
N1—C5	1.359 (3)	C7—H7	0.9300
N1—C1	1.370 (3)	C9—C8	1.406 (4)
N1—H1	0.8600	C9—H9	0.9300
O2—C6	1.272 (3)	C4—H4	0.9300
C6—N2	1.368 (3)	C8—H8	0.9300
			
O1—Ag—O1^i^	180	C6—O2—Ag	125.40 (14)
O1—Ag—O2	91.09 (5)	O2—C6—N2	118.79 (19)
O1—Ag—O2^i^	88.91 (5)	O2—C6—C7	125.1 (2)
O1—Ag—O2^ii^	89.50 (5)	N2—C6—C7	116.14 (19)
O1—Ag—O2^iii^	90.50 (5)	C10—N2—C6	123.64 (19)
O1^i^—Ag—O2	88.91 (5)	C10—N2—H2	118.2
O1^i^—Ag—O2^i^	91.09 (5)	C6—N2—H2	118.2
O1^i^—Ag—O2^ii^	90.50 (5)	O1—C1—N1	119.33 (19)
O1^i^—Ag—O2^iii^	89.50 (5)	O1—C1—C2	124.96 (19)
O2—Ag—O2^i^	180	N1—C1—C2	115.70 (18)
O2—Ag—O2^ii^	89.18 (5)	N1—C5—C4	120.4 (2)
O2—Ag—O2^iii^	90.82 (5)	N1—C5—H5	119.8
O2^i^—Ag—O2^ii^	90.82 (5)	C4—C5—H5	119.8
O2^i^—Ag—O2^iii^	89.18 (5)	C9—C10—N2	120.7 (2)
O2^ii^—Ag—O2^iii^	180	C9—C10—H10	119.7
F1—P1—F1^iv^	179.43 (13)	N2—C10—H10	119.7
F1—P1—F3^iv^	90.33 (8)	C3—C2—C1	120.7 (2)
F1^iv^—P1—F3^iv^	90.07 (8)	C3—C2—H2A	119.7
F1—P1—F3	90.07 (8)	C1—C2—H2A	119.7
F1^iv^—P1—F3	90.33 (8)	C2—C3—C4	120.8 (2)
F3^iv^—P1—F3	90.26 (12)	C2—C3—H3	119.6
F1—P1—F2^iv^	89.82 (8)	C4—C3—H3	119.6
F1^iv^—P1—F2^iv^	89.78 (8)	C8—C7—C6	120.3 (2)
F3^iv^—P1—F2^iv^	90.03 (8)	C8—C7—H7	119.9
F3—P1—F2^iv^	179.68 (9)	C6—C7—H7	119.9
F1—P1—F2	89.78 (8)	C10—C9—C8	118.0 (2)
F1^iv^—P1—F2	89.82 (8)	C10—C9—H9	121.0
F3^iv^—P1—F2	179.69 (9)	C8—C9—H9	121.0
F3—P1—F2	90.03 (8)	C5—C4—C3	118.5 (2)
F2^iv^—P1—F2	89.67 (12)	C5—C4—H4	120.7
C1—O1—Ag	130.04 (14)	C3—C4—H4	120.7
C5—N1—C1	123.91 (18)	C7—C8—C9	121.3 (2)
C5—N1—H1	118.0	C7—C8—H8	119.4
C1—N1—H1	118.0	C9—C8—H8	119.4
			
O2^i^—Ag—O1—C1	170.23 (18)	C1—N1—C5—C4	−0.7 (3)
O2—Ag—O1—C1	−9.77 (18)	C6—N2—C10—C9	−0.4 (3)
O1^i^—Ag—O2—C6	−28.01 (17)	O1—C1—C2—C3	−178.6 (2)
O1—Ag—O2—C6	151.99 (17)	N1—C1—C2—C3	1.5 (3)
Ag—O2—C6—N2	20.6 (3)	C1—C2—C3—C4	−0.7 (4)
Ag—O2—C6—C7	−160.21 (16)	O2—C6—C7—C8	−178.1 (2)
O2—C6—N2—C10	178.9 (2)	N2—C6—C7—C8	1.1 (3)
C7—C6—N2—C10	−0.4 (3)	N2—C10—C9—C8	0.5 (3)
Ag—O1—C1—N1	3.3 (3)	N1—C5—C4—C3	1.5 (3)
Ag—O1—C1—C2	−176.67 (15)	C2—C3—C4—C5	−0.8 (4)
C5—N1—C1—O1	179.28 (19)	C6—C7—C8—C9	−1.1 (4)
C5—N1—C1—C2	−0.8 (3)	C10—C9—C8—C7	0.2 (4)

Symmetry codes: (i) −*x*, −*y*, −*z*; (ii) −*x*, *y*, −*z*+1/2; (iii) *x*, −*y*, *z*−1/2; (iv) −*x*−1, *y*, −*z*+1/2.

### Hydrogen-bond geometry (Å, °)

**Table:**

*D*—H···*A*	*D*—H	H···*A*	*D*···*A*	*D*—H···*A*
N1—H1···O2	0.86	1.91	2.765 (2)	171
N2—H2···O1^v^	0.86	1.90	2.754 (3)	174
C3—H3···F1^vi^	0.93	2.48	3.353 (3)	157
C5—H5···F3^iv^	0.93	2.51	3.398 (3)	159

Symmetry codes:  (v) −*x*, −*y*, −*z*; (vi) *x*+1/2, −*y*+1/2, *z*−1/2; (iv) −*x*−1, *y*, −*z*+1/2.
